# Tumor- and Osteoblast-Derived Periostin in Prostate Cancer bone Metastases

**DOI:** 10.3389/fonc.2021.795712

**Published:** 2022-01-11

**Authors:** Chuan-Yu Sun, Yuan-Yuan Mi, Sheng-Yang Ge, Qing-Feng Hu, Ke Xu, Yi-Jun Guo, Yi-Fan Tan, Yang Zhang, Fan Zhong, Guo-Wei Xia

**Affiliations:** ^1^ Department of Urology, Huashan Hospital, Fudan University, Shanghai, China; ^2^ Department of Urology, Affiliated Hospital of Jiangnan University, Wuxi, China; ^3^ Department of Urology, Jing’an District Central Hospital, Fudan University, Shanghai, China; ^4^ Department of Systems Biology for Medicine, Shanghai Medical College, Fudan University, Shanghai, China; ^5^ Institutes of Biomedical Sciences, Fudan University, Shanghai, China

**Keywords:** prostate cancer, bone metastasis, periostin, osteoblast, integrin receptor

## Abstract

Exploring the biological function of periostin (POSTN) in prostate cancer (PCa) bone metastasis is of importance. It was observed that the expression of POSTN was high in PCa, especially highest in PCa metastasized to bone. In this study, we found that inhibiting POSTN in PCa cells could significantly alleviate PCa bone metastasis *in vivo*, suggesting POSTN is a promising therapeutic target. Since, due to the secreted expression of POSTN in osteoblasts and PCa, we hypothesized the positive feedback loop between osteoblasts and PCa mediated by POSTN in PCa bone metastasis. The *in vitro* experiments demonstrated that osteoblast-derived POSTN promoted PCa cell proliferation and invasion and PCa cell-derived POSTN promotes proliferation of osteoblasts. Furthermore, we found that POSTN regulated PCa and osteoblast function through integrin receptors. Finally, ^18^F-Alfatide II was used as the molecule probe of integrin αvβ3 in PET-CT, revealing high intake in metastatic lesions. Our findings together indicate that targeting POSTN in PCa cells as well as in the osteoblastic may be an effective treatment for PCa bone metastasis.

## Introduction

Prostate cancer (PCa) cells possess a propensity to metastasize to bone (1, 2). Nearly all patients who die from PCa display evidence of bone metastasis (3). Although current therapies for patients with PCa bone metastasis such as androgen deprivation and chemotherapy can inhibit the progression of the disease, 90% of the patient will relapse within 1 year (4–6). Thus, new therapeutic molecular target(s) are urgently needed. It is well studied that cancer bone metastasis relies on the crosstalk between cancer cells and bone cells, providing a fertile “soil” for the settlement and growth of the “seeds” in bones (2, 7). It is important to explore the novel target(s) mediating the crosstalk between PCa and bone cells, particularly the matricellular proteins.

Transforming growth factor-β (TGF-β) plays a crucial role in controls many normal physiological processes, including embryogenesis, immune responses, and bone remodeling (8). In the study of cancer, TGF-β is reported to promote invasion and metastasis, in particular metastasis to bone (9). TGF-β is one of the most abundant growth factors in bone and is released during osteoclastic bone resorption. TGF-β signaling is activated in bone metastases samples from breast cancer (BCa) patients (10) and PCa bone metastasis (11). Mechanistically, TGF-β upregulates the integrin-mediated adhesion of PCa cells to bone-derived type I collagen to promote bone metastasis (12). Recently, increasing evidences indicated the role of integrin in bone metastasis. Briefly, integrin alpha(v)beta3 (αVβ_3_) is involved in bone metabolism and angiogenesis, pointing to the role of these processes in controlling growth of metastatic prostate cancer cells in the bone (13), which is confirmed by other studies (14, 15). Nowadays, αVβ_3_ is demonstrated as a therapeutic target for breast cancer bone metastases (16). These studies illustrated the role of TGF-β/integrin pathway in bone metastasis. However, how TGF-β regulates integrin-mediated bone metastasis remains unclear.

Periostin (POSTN) is originally identified from osteoblasts which is preferentially expressed in the periosteum (17, 18). As a matricellular protein, POSTN is mainly secreted into the extracellular matrix (ECM) and works as a nature ligand of the integrin family (19). Its upregulated expression is commonly displayed in a wide variety of cancers although it is commonly absent in most of normal tissues (20–22). POSTN is thought to be correlated with cell survival, metastasis, and epithelial-mesenchymal transition (EMT) of cancer cells (23, 24). Our previous study showed that POSTN was overexpressed in PCa cells and it was regulated by TGF-β to promote EMT (25). Further studies demonstrated the role of POSTN in breast cancer bone metastases (26) and lung cancer bone metastases (27).

The tumor microenvironment is crucial for bone metastasis (28). Especially, the matricellular proteins were reported to promote PCa bone metastasis (29, 30). Summarizing the previous results, we find that: (1) POSTN is identified in osteoblasts and preferentially expressed in the periosteum as a putative bone adhesion molecule (17, 18); (2) POSTN is a secreted matricellular protein (31); (3) POSTN was regulated by TGF-β (25); (4) POSTN is a ligand of the integrin family (19, 32); and (5) POSTN promotes EMT of PCa cells (33). These results together imply POSTN may be a mediator between TGF-β and integrin, which prompt us to explore the role of TGF-β/POSTN/integrin axis in PCa bone metastasis.

Herein, this study concentrated on the impact of endocrine of POSTN between local lesions of PCa and distant bone metastases after we previously investigated the mechanism of autocrine and paracrine of POSTN in PCa cells. We attempted to discover the evidence of POSTN related to metastasis in PCa through multiomics, corroborated these outcomes by the Cancer Genome Atlas (TCGA) database, figured out whether POSTN derived from PCa cells and osteoblasts functioned similarly, and sought for the peculiar role of POSTN in PCa bone metastasis by cellular experiment. Profoundly, we made use of high-efficient Alfatide II tracer ^18^F-NOTA-E[PEG_4_-c(RGDfk)]_2_ to promulgate the valid value in clinical cohort observation. Our findings indicate that targeting POSTN in prostate cancer cells as well as in osteoblast may be an effective treatment for PCa bone metastasis.

## Materials and Methods

### Orthogonal Projections to Latent Structures Discriminant Analysis

We utilized the TCGA (http://tcga-data.nci.nih.gov/tcga/) to obtain all the RNA-seq data of POSTN in PCa. We then divided these data into four independent groups by evaluation of clinical stages, following-up situation, and remote metastases: normal prostate cancer as control group (C group; 429 cases), local recurrence as the LR group (6 cases), biological recurrence as the BR group (53 cases), and distant metastasis as the DM group (5 cases). Orthogonal projections to latent structures discriminant analysis (OPLS-DA) was applied to analyze the difference between these four groups by dimensionality reduction into two main components.

### Volcano Plot of DM Group vs. Control Group

Significantly differentially expressed mRNAs of POSTN were identified through volcano plot filtering. In this volcano plot, the *x*-axis represents the fold change between two comparison groups (DM vs. C), and the *y*-axis represents the *p*-value by *t*-test.

### Relative mRNA Expression of POSTN in the DM Group vs. Control Group

To further specify the relative expression levels of POSTN in DM group and control group, relative mRNA expression levels were calculated by using normalized relative to the expression levels of POSTN.

### Oncomine Analysis

POSTN mRNA levels in prostate cancer tissues and normal tissues were determined using the Oncomine database (www.oncomine.org) (34). Relative expression values were transformed to log2(RPKM) representing relative expression levels.

### Survival Curve Analysis

We used the Gene Expression Profiling Interactive Analysis (GEPIA, http://gepia2.cancer-pku.cn/) as the analysis database to search for the disease-free survival (DFS) of POSTN (35).

### Orthogonal Transcriptional Regulation Network of POSTN in Proteomes

The networks of transcription regulation in the differentially expressed proteins were generated by MetaCore™ software through using a transcription regulation algorithm. Differentially expressed regulators in both upstream and downstream of POSTN were clued by ingenuity pathway analysis (IPA) (36). Genes involved went through a threshold of a ranking *p*-value of less than 0.01, and a log2 fold change of greater than 1 was considered significant. The complete description of the nodes and relationship in **Figure 3A** are illustrated at https://portal.genego.com/legends/MetaCoreQuickReferenceGuide.pdf. The full details of the shapes and arrows between nodes in **Figure 3B** are described at https://qiagen.secure.force.com/KnowledgeBase/articles/Basic_Technical_Q_A/Legend.

### Cell Culture

The human PCa cell lines (PC-3 and MDA PCa 2b) and hFOB 1.19 (hFOB) osteoblasts were purchased from American Type Culture Collection (Manassas, VA, USA). PC3 cells were maintained in RPMI-1640 medium (Thermo Fisher, Waltham, MA, USA) supplemented with 10% fetal bovine serum (FBS, Thermo Fisher, Waltham, MA, USA), 2 mM l-glutamine (Sigma-Aldrich, St. Louis, MO, USA), 100 U/ml penicillin, and 100 μg/ml streptomycin (both from Invitrogen, Carlsbad, CA, USA). MDA PCa 2b cells were maintained in Ham’s F-12K medium (Thermo Fisher, Waltham, MA, USA) containing 20% FBS, 25 ng/ml cholera toxin (Sigma-Aldrich, St. Louis, MO, USA), 10 ng/ml mouse epidermal growth factor (Corning, Corning, NY, USA), 0.005 mM phosphoethanolamine, 100 pg/ml hydrocortisone, 45 nM sodium selenite, and 0.005 mg/ml human recombinant insulin (all from Sigma-Aldrich, St. Louis, MO, USA). Both PC cell lines were cultured at 37°C in a humidified incubator with 5% CO_2_. hFOB cells were maintained in DMEM/F-12 Medium (Thermo Fisher, Waltham, MA, USA) supplemented with 10% FBS, 2.5 mM l-glutamine, and 0.3 mg/ml G418 (Sigma-Aldrich, St. Louis, MO, USA) at 34°C in a humidified incubator with 5% CO_2_.

To obtain osteoblast- or PCa cell-conditioned medium (OBCM or PCCM), cells were grown to confluence and the culture medium was changed to base medium without FBS. OBCM or PCCM was collected 48 h later and stored at −70°C until use.

### Cell Proliferation Assays

MDA PCa 2b cells or hFOB cells were seeded at 2.0 × 104 cells/ml in 96-well plates. The medium was then replaced with complete medium and indicated supplement. After 72 h culture, cell proliferation assays were performed using the CellTiter 96^®^ Non-Radioactive Cell Proliferation Assay kit according to the manufacturer’s instruction (Promega, Madison, WI, USA).

### Gene Knockdown by RNA Interference

The knockdown of POSTN gene expression was performed by siRNA transfection. PCa cells or hFOB cells were grown to 80% confluence in 6-well plates and then transfected with lentiviral particles containing POSTN shRNA (Lv-POSTN shRNA; Santa Cruz, Dallas, TX, USA) (sc-61324-V) according to the manufacturer’s instructions. Briefly, PCa cells or hFOB cells were plated in a 12-well plate and incubated to approximately 50% confluent. Complete medium was then replaced with Polybrene (5 µg/ml) (Santa Cruz)/media mixture, and 20 µl Lv-POSTN shRNA was added. After 24 h, the culture medium was removed and replace with 1 ml of complete medium. To select stable clones expressing the shRNA, split cells 1:3, and continue incubating for 48 h in complete medium. A total of 5 µg/ml puromycin was added into medium, and the medium was replaced every 3–4 days until resistant colonies can be identified. The silence of POSTN expression was verified by real-time PCR and Western blotting.

### Real-Time PCR

Total RNA was extracted from cells using TRIzol reagent (Invitrogen), and reverse transcription reactions were performed to synthesize cDNA by using a reverse transcription kit (Thermo Fisher, Waltham, MA, USA) according to the manufacturer’s protocols. Quantitative real-time PCR was carried out using SYBR green supermix (Tiangen, Beijing, China) with primers from Applied Biosystems StepOnePlus system (Foster City, CA, USA) as described previously (37). The results were normalized to the expression of glyceraldehyde-3-phosphate dehydrogenase (GAPDH) and displayed as relative expression compared with the control.

### Western Blotting

After washing twice with cold phosphate buffer saline (PBS), cells were lysed with RIPA lysis buffer (Beyotime, Haimen, China) containing protease inhibitor. Protein concentration was quantified using the BCA Protein Assay kit (Beyotime, Haimen, China). After separation by SDS-PAGE, the proteins were transferred onto Immobilon polyvinyldifluoride membranes. Blots were blocked with 4% bovine serum albumin in Tris-buffered saline and probed with anti-POSTN or GAPDH polyclonal antibodies (Abcam, Cambridge, UK) overnight at 4°C. After washing, the blots were incubated with secondary antibody for 1 h at room temperature. Blots were visualized using an enhanced chemiluminescence detection system (Cell Signaling, Beverly, MA, USA).

### Enzyme-Linked Immunosorbent Assay

Control or experimental MDA PCa 2b and hFOB cells stably transfected with Lv-POSTN shRNA were grown to confluence and culture media were changed to base medium without FBS. After 48 h, secreted POSTN was measured using an enzyme-linked immunosorbent assay (ELISA) kit (R&D Systems, Minneapolis, MN, USA) according to the manufacturer’s instructions.

### Invasion Assay

Cell invasive ability was detected by using the QCM Collagen Cell Invasion Assay kit (24-well, 8 µm; Millipore, Burlington, MA, USA). Briefly, cells were cultured with the indicated medium for 48 h and pretreated with monoclonal antibodies against POSTN, αVβ_3_, or αVβ_5_ (all from Abcam) for 1 h. After harvesting in serum-free medium, 5 × 104 cells were placed in the upper chamber, while 300 μl of medium containing 10% FBS and OBCM or PCCM were added to the lower chamber. After incubating for 24 h at 37°C in 5% CO_2_, cells on the upper side of the filters were removed with cotton-tipped swabs and invasive cells were stained using staining solution. Cells were counted in six random fields under a microscope. Each experiment was repeated at least three times.

### Immunofluorescence Staining

MDA PCa 2b cells and hFOB cells were seeded on glass cover slips and fixed with 4% paraformaldehyde in PBS containing 0.05% Triton X-100 and 1% goat serum (Invitrogen) for 30 min. The cells were then incubated with anti-αVβ_3_ or αVβ_5_ antibodies (Abcam) overnight at 4°C. After washing, the cells were incubated with Alexa Fluor 488-conjugated secondary antibody (Abcam) for 1 h at room temperature, then counterstained with 4′,6-diamidino-2-phenylindole dihydrochloride (DAPI) solution (Sigma-Aldrich). Slips were observed and imaged under a fluorescence microscope.

### Orthotopic Bone Tumor Model in Nude Mice

An orthotopic bone tumor model of metastatic PCa was constructed using 6–8-week-old male nude mice (nu/nu) according to Park et al. (38). Briefly, stable luciferase-expressing MDA PCa 2b cells (MDA PCa 2b-luc) were transfected with Lv-POSTN shRNA and stable clones (MDA PCa 2b-luc-POSTN shRNA) were selected. Mice were anesthetized, and 10 μl of MDA PCa 2b-luc cells (1 × 10^6^) or MDA PCa 2b-luc-POSTN shRNA cells were injected into the tibia. PBS was injected into the sham group. Bioluminescence imaging was performed every week for 5 weeks using the IVIS 200 Imaging System and Living Image software (Caliper Life Sciences, Hopkinton, MA, USA). Approximately 10–15 min before each imaging, the mice were injected i.p. with 150 mg/kg d-luciferin (Biosynth, Staad, Switzerland).

### Bone Metastasis Assays in Nude Mice

MDA PCa 2b-luc cells or MDA PCa 2b-luc-POSTN shRNA cells (1 × 10^6^ cells in 100 μl) were injected into the left ventricle of 6–8-week-old male nude mice (nu/nu) to study the bone metastasis activity according to Park et al. (38). Bioluminescence imaging was performed every week for 60 days to quantify the metastasis burden at the target organs using IVIS 200 Imaging System and Living Image software. Metastasis-free (no metastasis to any organs) survival curve was drawn by SPSS 13.0. All animal studies were approved by the Animal Care and Use Committee of Fudan University.

### The Recruitment of Patient Cohorts for ^18^F-Alfatide II PET/CT Examination

Twenty-one patients with PCa were recruited and were detected by ^18^F-Alfatide II PET/CT examination. The final diagnosis of primary and bone lesions was established based on the comprehensive assessment of all clinical data and follow up. Our current clinical examination was approved by the ethics committee of the Affiliated Hospital of Jiangnan University.

### PET/CT Protocol and Image Analysis

^18^F-Alfatide II PET images were scored according to the visual analysis on a per-lesion basis by two senior nuclear medicine physicians who were unaware of the clinical diagnosis and reached consensus. A 4-point grade system was espoused to define the intake degree of bone lesions, as illustrated in **Table 1**. Grade 0 lesions were recognized as negative lesions, and lesions with a score greater than grade 0 were characterized as positive lesions. The final diagnosis was based on clinical follow-up and imaging results. Differential diagnosis was made with the following diseases: bone inflammatory diseases like osteomyelitis, sarcoidosis and tuberculosis, bone fracture, and other bone trauma within 6 months. Bone metastasis was diagnosed by matching any of the following criteria: a score equaled to or greater than grade 2 on ^18^F-Alfatide II PET, a score equaled to grade 1 on ^18^F-Alfatide II PET, or a lesion confirmed on ^99^mTc-MDP bone scan, X-ray, CT (including the corresponding CT of PET/CT), or MRI examination. The osteolytic, osteoblastic, mixed bone metastatic lesions were classified based on CT characteristics (39, 40).

**Table 1:** The 4-point grade system for scoring the intake degree of bone lesions.

Grade	Detailed description
0	Intake similar to the surrounding bone structure
1	Slightly higher intake than the surrounding bone structure
2	Significantly higher intake than the surrounding bone structure
3	Abnormal concentrated intake

### Statistical Analysis

Data were presented as means ± standard error of mean. Statistical significance was assessed by Student’s *t*-test and one-way analysis of variance followed by Tukey’s *post-hoc* test. The Kaplan-Meier method was used for bone metastasis-free survival. *p* < 0.05 was considered statistically significant.

### Ethical Review

All participants provided written informed consent to take part in the study, and this project was permitted by Huashan Institutional Review Board (HIRB) Approval Letter. The approval number was (2011)009.

## Result

### POSTN Is a Potential Therapeutic Target in Treating PCa Bone Metastasis

#### POSTN Is Highly Expressed in PCa Bone Metastasis

**Figure 1:**
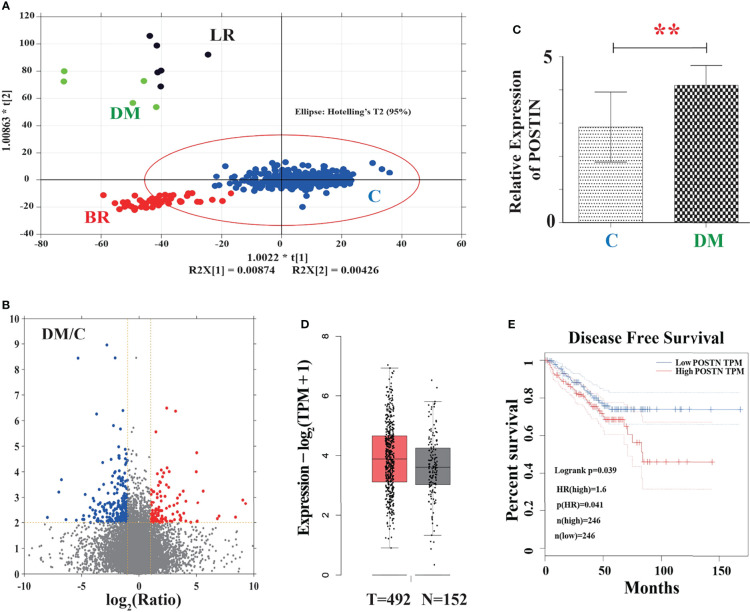
The association between POSTN and metastasis in PCa was verified by the TGCA database. **(A)** The outcome of OPLS-DA. C, control; LR, local recurrence; BR, biochemical recurrence; DM, distant metastases. In this figure, the points inside the red line spheroid means a least significant difference test at a 95% confidence level. **(B)** Volcano plot showing differentially expressed proteins. Red color represents a high relative expression level; blue color represents a low relative expression level. **(C)** Relative mRNA expression of POSTN in the DM group vs. control group. ^**^ *p* < 0.01. **(D)** POSTN expression between normal and patients verified by the Oncomine database. **(E)** Survival curve analysis of disease-free survival (DFS). DFS time between higher-expression level and lower-expression level of POSTN in PCa. Red line displays the cases with highly expressed POSTN, and blue line reveals the cases with lowly expressed POSTN. HR, hazard ratio. *p*(HR) = 0.041.

To address whether POSTN is highly expressed in PCa bone metastasis, a systematic analysis was performed based on the TCGA database and Oncomine dataset. As shown in **Figure 1A**, the outcome of OPLS-DA exhibited that the group of DM and LR were obviously dispersed from the control group and part of BR group while the group of DM and LR kept a long distance from each other, suggesting that there was a great difference in mRNA expression between control and DM. In the volcano plot (**Figure 1B**), there was a good consistency between upregulated and downregulated proteins of PCa in the TCGA database. In total, 859 differentially expressed genes were captured, among of them, 345 were upregulated and 514 were downregulated. Furthermore, we selected the expression data of POSTN and found that the relative mRNA expression of POSTN in DM group was significantly higher than that in the control group (*p* < 0.01) (**Figure 1C**). On the contrary, we found there may be no significant difference between these two groups by analyzing the data of PCa tissues (492 cases) and normal tissues (152 cases) from the Oncomine dataset (**Figure 1D**). According to the definition of disease-free survival time “since the patients who maintain disease free for an extensive period of time may be cured”, the data demonstrated that the overexpression of POSTN led to a shorter time to recurrence in the PCa (**Figure 1E**). These results suggest POSTN may be a prometastasis protein in PCa and high expression of POSTN indicates shorter time to recurrence.

#### Targeting POSTN Effectively Alleviates Bone Metastasis *In Vivo*

**Figure 2:**
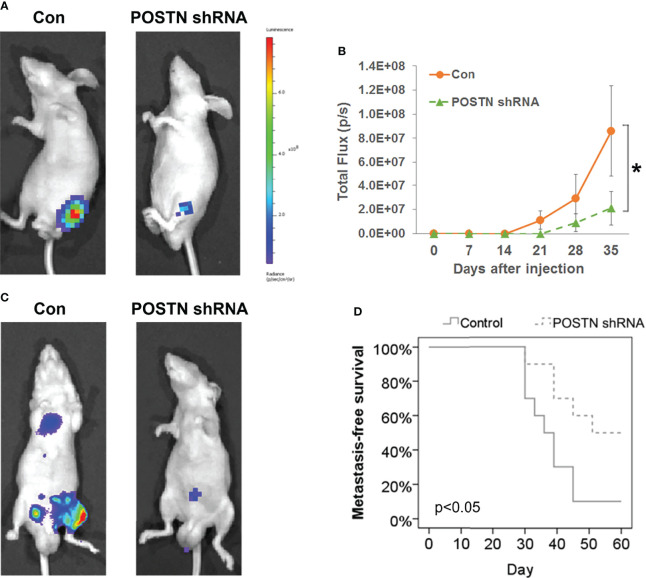
POSTN knockdown suppresses the bone metastasis capability of PCa cells. **(A)** MDA PCa 2b cells or POSTN-knockdown MDA PCa 2b cells were injected in the intramedullary cavity of the tibia. Shown are representative whole-animal luciferase imaging of mice 5 weeks postsurgery (*n* = 8/group). **(B)** Bioluminescent curve of tumor development [related to **(A)**]. **(C)** MDA PCa 2b cells or POSTN-knockdown MDA PCa 2b cells were injected into the left ventricle in nude mice. Representative whole-animal luciferase imaging of mice 60 days postsurgery (*n* = 8/group). **(D)** Metastasis-free survival [related to **(C)**]. ^*^ *p* < 0.05.

Our previous *in vitro* studies showed the prometastasis role of POSTN *via* EMT (33). Since we have found that POSTN is highly expressed in PCa bone metastasis, we hypothesized that inhibiting POSTN in PCa cells will inhibit PCa bone metastasis. We therefore tested whether depletion of POSTN would suppress the bone metastasis capability of PCa cells in the orthotopic bone tumor model by implantation of tumor cells into tibia of mice. Whole-animal imaging showed that the luciferase activity in POSTN knockdown cells were lower than that in the control group (**Figure 2A**) and the quantitation of the bioluminescent signal displayed a markedly lower growth rate in the POSTN knockdown cells than in the control cells (**Figure 2B**). Furthermore, we successively evaluated the contribution of POSTN to bone metastasis of PCa cells by intracardiac injection of tumor cells in mice. As expected, the inhibition of POSTN in PCa cells lead to a significant reduced cells metastasis to bone (**Figure 2C**) and a significant improvement in metastasis-free survival (**Figure 2D**). These data cumulatively suggest that inhibiting POSTN in PCa cells effective against the metastasis of PCa cells to bone.

### The High Confidence Networks Constructed by Multiomics Revealed the Role of TGF-β1/POSTN/Integrin αvβ3 in PCa Bone Metastases

**Figure 3:**
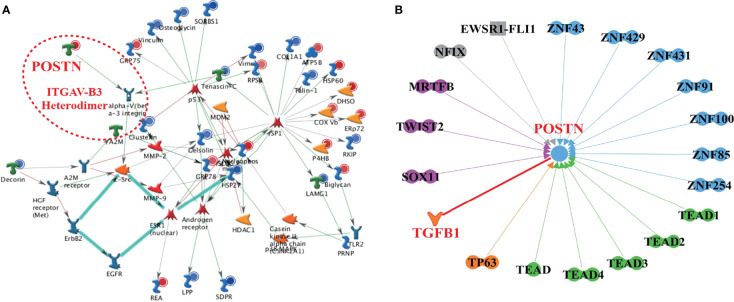
The high-confidence networks of POSTN were constructed by multiomics. **(A)** The top 1 enrichment network of Metacore™. The ITGAV-B3 heterodimer presented integrin αVβ3. **(B)** The interaction network of POSTN by IPA. Dumbbell shape: transcription regulator; boomerang shape: growth factor; the arrow direction: activating.

To explore the underlying mechanism of POSTN in PCa bone metastases, a high confidence network was constructed by multiomics. From the top 1 enrichment network of Metacore™, we found that the integrin αvβ_3_ was activated by POSTN as the ligand (**Figure 3A**). Both of these molecules were significantly upregulated. Through the combination of POSTN and intergrin αvβ_3_, the signaling pathway of c-Src is directly influenced, which was proved to regulate the cell survival and invasion in osteoblast (41). In **Figure 3B**, the interaction network demonstrated almost all the known proteins related to POSTN. POSTN was revealed to get activated by the family of transcriptional-enhanced associate domain (TEAD) protein, the family of zinc finger (ZNF) protein, TGF-β1, tumor protein p63 (TP63), SRY-box transcription factor 11 (SOX11), twist family bHLH transcription factor 2 (TWIST2), myocardin-related transcription factor B (MRTFB), nuclear factor I X (NFIX), and fetal and adult testis-expressed transcript protein (EWSR1-FLI1), which hinted the possible upstream regulators of POSTN. Among them, only TGF-β1 was a growth factor while the others were transcription regulators. These proteins were involved in the tumor survival, proliferation, invasion, and metastasis. Together, the data promote us to focus on the role of TGF-β1/POSTN/integrin αvβ3 axis in PCa bone metastases.

### POSTN Is a Mediator in the Crosstalk Between PCa Cells and Osteoblasts

Based on the networks constructed by multiomics, TGF-β1 is a key factor regulating POSTN expression, which is reported in our previous study (33) and conformed in this study (**Supplementary Figure S1**). Herein, we focused on the role of POSTN in the crosstalk between PCa cells and osteoblasts.

#### Osteoblast-Derived POSTN Promotes PCa Cell Proliferation and Invasion

**Figure 4:**
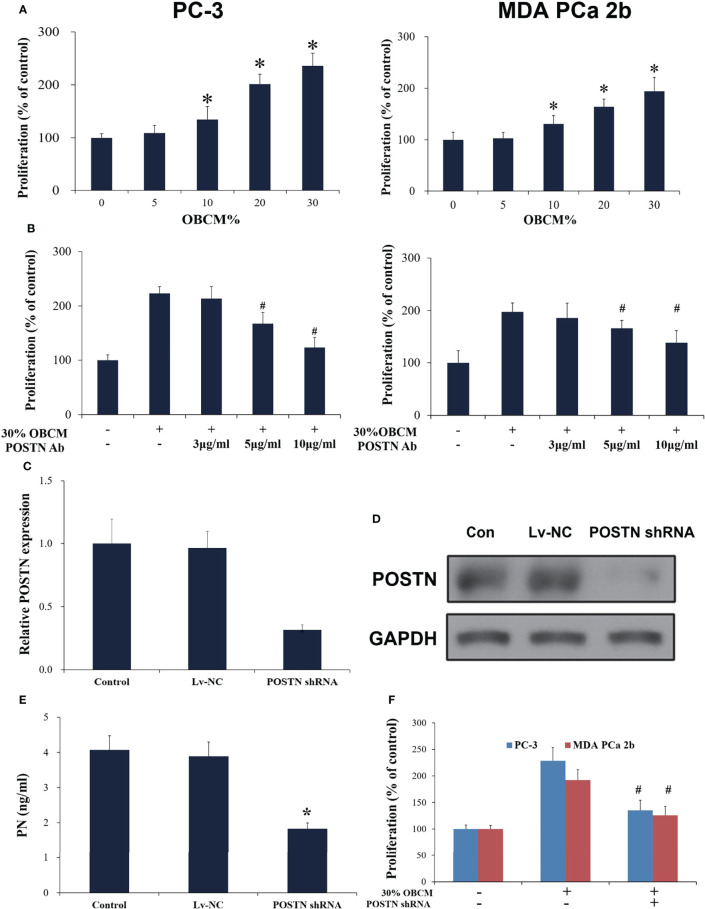
Osteoblast-derived POSTN promoted proliferation of PCa cells. **(A)** PC-3 and MDA PCa 2b cells were incubated with various concentrations of osteoblast (hFOB)-conditioned medium (OBCM) for 48 h before proliferation was assessed. **(B)** PC-3 and MDA PCa 2b cells were incubated with 30% OBCM supplemented with various concentrations of POSTN monoclonal antibody for 48 h before proliferation was assessed. **(C)** hFOB cells were transfected with control shRNA lentivirus (Lv-NC) or POSTN shRNA lentivirus (POSTN shRNA). The expression of POSTN was detected by real-time PCR. **(D)** hFOB cells were transfected with control shRNA lentivirus (Lv-NC) or POSTN shRNA lentivirus (POSTN shRNA). The expression of POSTN was detected by Western blotting. **(E)** The levels of POSTN in hFOB cell medium were detected by ELISA. **(F)** PC-3 and MDA PCa 2b cells were incubated with 30% OBCM from control or POSTN-knockdown hFOB cells. Proliferation was assessed after 48 h. ^*^ *p* < 0.05 compared with control; ^#^ *p* < 0.05 compared with the OBCM-treated group.

**Figure 5:**
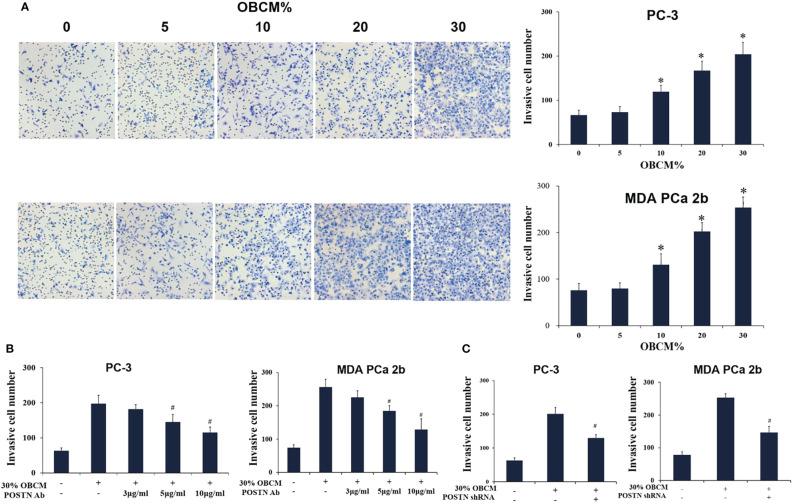
Osteoblast-derived POSTN promotes invasion of PCa cells. **(A)** PC-3 and MDA PCa 2b cells were incubated with various concentrations of osteoblast (hFOB)-conditioned medium (OBCM), and cell invasion was measured by Transwell^®^ assays. **(B)** PC-3 and MDA PCa 2b cells were incubated with 30% OBCM supplemented with various concentrations of POSTN monoclonal antibody, and cell invasion was measured by Transwell^®^ assays. **(C)** PC-3 and MDA PCa 2b cells were incubated with 30% OBCM from control or POSTN-knockdown hFOB cells. Cell invasion was measured by Transwell^®^ assays. ^*^ *p* < 0.05 compared with the control; ^#^ *p* < 0.05 compared with the OBCM-treated group.

To mimic the effect of soluble components in the bone-derived microenvironment, osteoblast-conditioned medium (OBCM) was utilized for PCa cells. We found that the OBCM promoted the proliferation of PC-3 and MDA PCa 2b cells, and the pro-proliferation effect of OBCM on PCa cells showed a concentration-dependent manner (**Figure 4A**). To further discover the contribution of POSTN in OBCM to PCa cell proliferation, POSTN signaling was blocked by a monoclonal antibody against POSTN (POSTN Ab). The results showed that adding POSTN Ab (5 and 10 μg/ml) significantly suppressed OBCM-promoted PCa cell proliferation (**Figure 4B**), indicating the proliferation effect of POSTN in OBCM. We then knocked down the expression of POSTN in osteoblast hFOB cells using lentivirus-mediated POSTN shRNA; the results showed POSTN shRNA could significantly inhibit the expression of POSTN at mRNA and protein level (**Figures 4C, D**). ELISA assay showed that POSTN concentration in serum-free medium from hFOB cells was also reduced by POSTN knockdown (**Figure 4E**). Moreover, the OBCM from the hFOB cells after POSTN shRNA transfection was used to treat PCa cells. We found the OBCM with low concentration of POSTN lost most of its proliferative activity (**Figure 4F**). To test the function of POSTN on invasion, Transwell^®^ assay was performed. The results revealed that the number of invaded PCa cells was increased in high concentrations of OBCM (10%–30%), showing a concentration-dependent manner (**Figure 5A**). When POSTN Ab and POSTN knockdown were used to reduce the concentration of POSTN in OBCM, the proinvasion function of OBCM on PCa cells was decreased (**Figures 5B, C**). These results indicated that osteoblast-derived POSTN promoted the proliferation and invasion of PCa cells.

#### PCa Cell-Derived POSTN Promotes Proliferation of Osteoblasts

**Figure 6:**
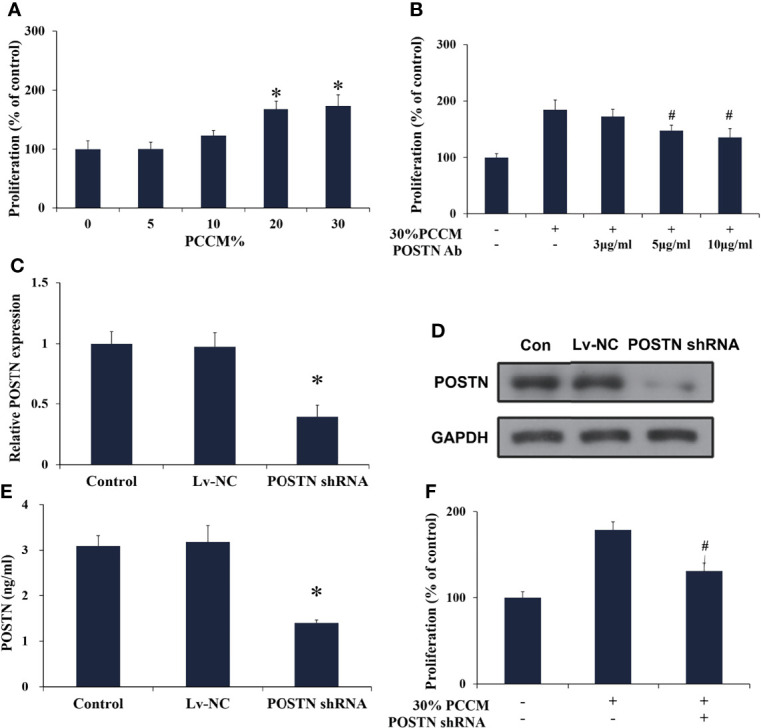
PCa cell-derived POSTN promotes proliferation of osteoblasts. **(A)** hFOB cells were incubated with various concentrations of PC cell (MDA PCa 2b)-conditioned medium (PCCM) for 48 h and proliferation was assessed. **(B)** hFOB cells were incubated with 30% PCCM supplemented with various concentrations of POSTN monoclonal antibody for 48 h, and proliferation was assessed. **(C)** MDA PCa 2b cells were transfected with control shRNA lentivirus (Lv-NC) or POSTN shRNA lentivirus (POSTN shRNA). The expression of POSTN was detected by real-time PCR. **(D)** MDA PCa 2b cells were transfected with control shRNA lentivirus (Lv-NC) or POSTN shRNA lentivirus (POSTN shRNA). The expression of POSTN was detected by Western blotting. **(E)** The levels of POSTN in MDA PCa 2b cell medium were detected by ELISA. **(F)** hFOB cells were incubated with 30% PCCM from control or POSTN-knockdown MDA PCa 2b cells. Proliferation was assessed after 48 h. ^*^ *p* < 0.05 compared with the control; ^#^ *p* < 0.05 compared with the OBCM-treated group.

We subsequently applied conditioned medium from MDA PCa 2b cells (PCCM) to identify the effect that PCa cell-derived microenvironment contributed to osteoblasts in the similar liquid nutrient concentration. We found that 20% and 30% PCCM incubation significantly promoted hFOB cell proliferation in a concentration-dependent manner (**Figure 6A**). Once POSTN was blocked by POSTN Ab, the pro-proliferation effect of PCCM was reduced (**Figure 6B**). Using the lentivirus-mediated POSTN shRNA, we reduced POSTN expression in MDA PCa 2b cells at mRNA (**Figure 6C**) and protein level (**Figure 6D**), as well as the concentrations of POSTN in PCCM (**Figure 6E**). We found that inhibition of POSTN in PCCM suppressed PCCM-promoted osteoblast proliferation (**Figure 6F**). These results implied that PCa cell-derived POSTN promoted proliferation of osteoblasts.

### POSTN Functions in PCa Cells and Osteoblasts *via* Integrin Receptors

**Figure 7:**
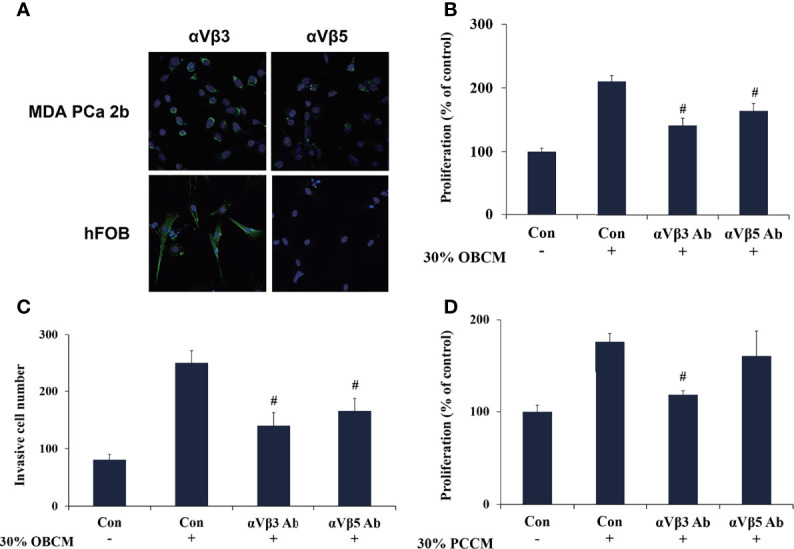
POSTN functions in PCa cells and osteoblasts *via* integrin receptors. **(A)** Immunofluorescence staining of αVβ3 and αVβ5 in MDA PCa 2b and hFOB cells. **(B)** MDA PCa 2b cells were incubated with 30% OBCM and αVβ3 or αVβ5 antibody. Cell proliferation was assessed. **(C)** MDA PCa 2b cells were incubated with 30% OBCM and αVβ3 or αVβ5 antibody. Invasions were assessed. **(D)** hFOB cells were incubated with 30% PCCM and αVβ3 or αVβ5 antibody. Cell proliferation was assessed. ^#^ *p* < 0.05 compared with the OBCM- or PCCM-treated group.

POSTN is reported to regulate cell function by binding to integrin receptors (23). Therefore, we investigated whether integrins mediated the interaction between PCa cells and osteoblasts. We assessed the expression of integrins αVβ_3_ and αVβ_5_, two major integrins mediating POSTN functions, in MDA PCa 2b cells and hFOB cells (42). Immunofluorescence assays suggested that αVβ_3_ was expressed in both MDA PCa 2b cells and hFOB cells, whereas αVβ_5_ was only weakly expressed in MDA PCa 2b cells and was rarely expressed in hFOB cells (**Figure 7A**). Specific neutralizing antibodies were then used to block integrin αVβ_3_ and αVβ_5_. The results presented that OBCM-enhanced cell proliferation and invasion in PCa cells were abrogated by either integrin αVβ_3_ or αVβ_5_ antibody (**Figures 7B, C**) while PCCM-enhanced cell proliferation in osteoblasts was attenuated by integrin αVβ_3_ antibody (**Figure 7D**). These data together proposed that the interaction of PCa cells and osteoblasts was probably mediated by POSTN and its integrin receptors.

### ^18^F-Alfatide II as the Molecule Probe of Integrin αvβ_3_ Revealed High Intake in Metastatic Lesions Through the PET-CT Detection

**Figure 8:**
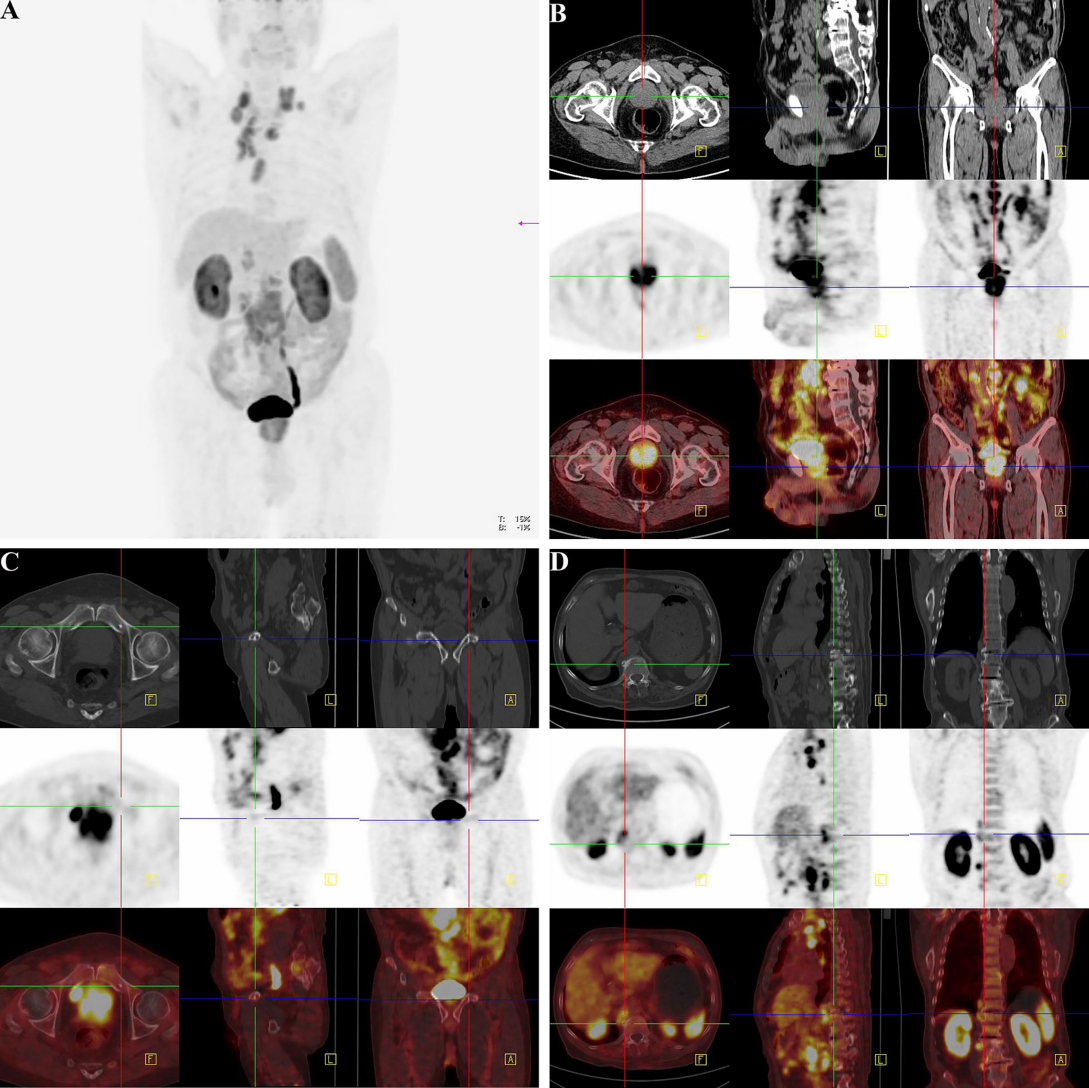
^18^F-Alfatide II as the molecule probe of integrin αvβ_3_ revealed high intake in primary and metastatic lesions through the PET-CT detection in a case of 82-year-old man. **(A)** Gross appearance of coronary position detected by PET-CT. The darker the color was, the higher the intake of tracer in the corresponding body part. **(B)** The direction cross focused on the primary lesion in prostate. The first row were CT views centered on high intake lesion of PCa from three different directions in the pelvis, which revealed the adjacent organs and structure. The second row were the PET-CT views detected by ^18^F-Alfatide II as the molecule probe of integrin αvβ_3_ centered on high intake lesion of PCa from three different directions. As the main function of bladder should be storing urine and the tracer was discharged by urine, it was rational that there was darker high-intake region in the bladder. However, the abnormal area of high-intake tracer appearing in the prostate suggested high-level affinity of probe which might be closely correlated to malignant cancer. The third row was combined with the figures of the first and second rows. The lighter the color shows the higher the intake of tracer in prostate. **(C)** The direction cross focused on the metastatic lesion in the middle part of the pubis from three differential directions. The first row was the view of regular CT, which revealed the bone structure and surrounding tissues. There was an unusual small spot of osteoproliferation in the direction cross. The second row demonstrated the abnormal high-intake lesion of ^18^F-Alfatide II in the left side of the middle part of the pubis, respectively, which was highly coincident with the “unusual small spot”, The third row was the combination of the first and second row, which systematically reported the accurate location of metastatic lesion in pubis and relative extent of tracer intaking. **(D)** The direction cross focused on the metastatic lesion on the right side of the lumbar 1 vertebra. The first row was regular CT view in the slice of abdomen. Compared with the adjacent vertebrae, the direction cross revealed high density in bone cortex, which was consistent with the characteristics of osteoblastic metastasis in PCa. In the second row, from the sight of ^18^F-Alfatide II, except for the rich blood supply organs like kidneys, spleen, and liver, the strange high intake of probe in the vertebra presented metastatic lesion. In the third row, the emerge of both first and second rows in the same slice then provided the reliable evidence of metastatic lesion form the aspects of position and signal strength.

**Figure 9:**
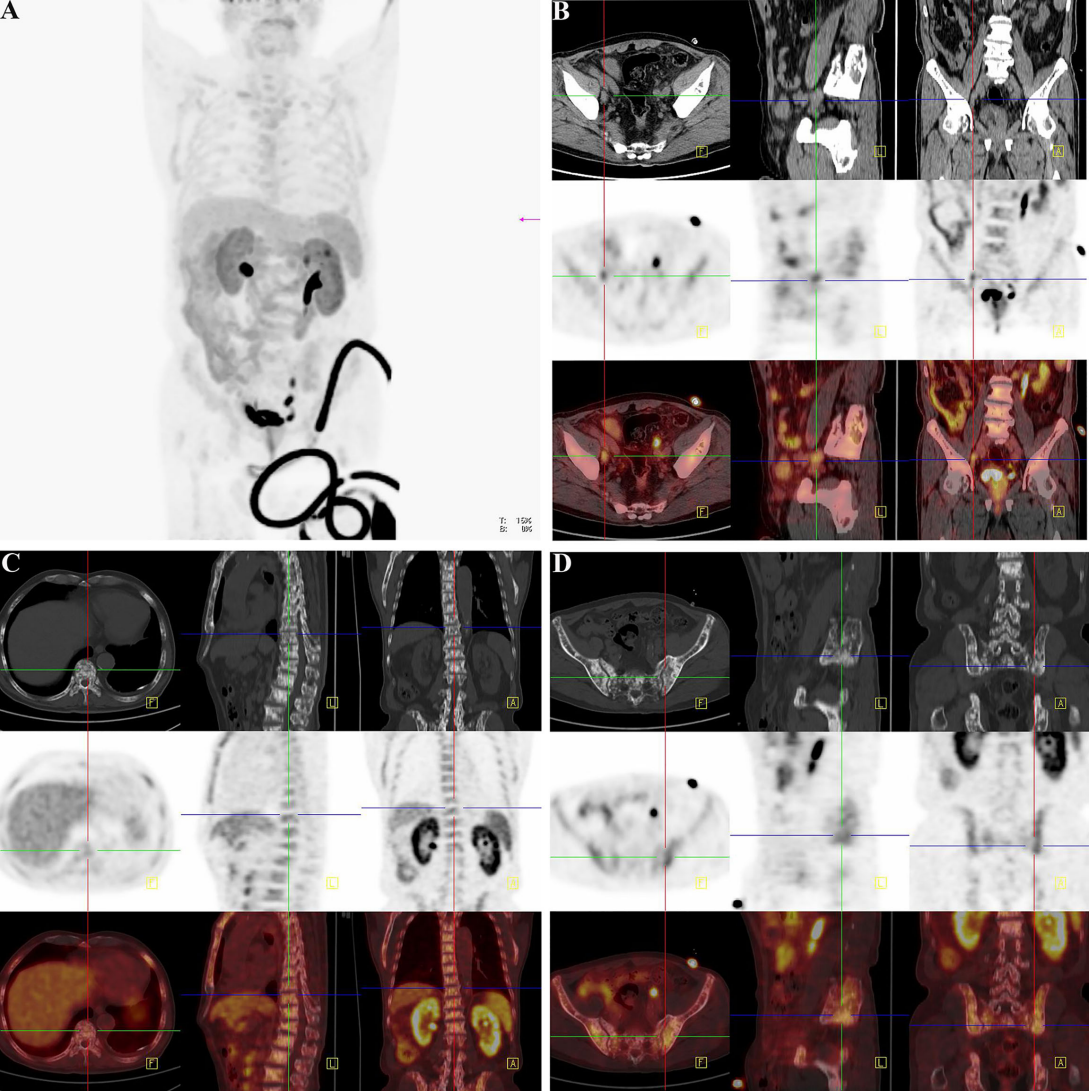
Another clinical replicate case from PET-CT in the case of 76-year-old man. **(A)** Gross view of coronary position detected by PET-CT. Similarly, the darker the color was, the higher the intake of tracer in the corresponding body part. Since the tracer was excreted through urine, the organs of urinary system were perceptibly high take of tracer. However, the prostate indicated abnormal high intake with dark tracer accumulation, consistent with local lesion of PCa. At the lower right corner of the picture, the spinning black “wire” should be the indwelling urine catheter and the urine with tracer inside. The bladder of the patient was not full, for the existence of urine catheter led the urine into the urine collection bag instantly. **(B)** The direction cross focused on the enlarged lymph node on the right side of the external iliac vessel. From the view of regular CT, the first row exhibited enlarged lymph node in right side of the external iliac vessel with the same density of soft tissue, and it displayed its neighboring tissues and bone structure. The specific nature of enlarged lymph node was unclear. The high-intake lesion on the right side of the external iliac vessel was also detected by the probe of ^18^F-Alfatide II in the second row from three directions, which suggested convincing evidence of lymphatic metastasis of PCa. Meanwhile, there were other two high intake points in the same slice of axial direction. As the association of the first and second rows, the third row demonstrated the highlighting point on the right side of the external iliac vessel was enlarged lymph node of metastasis while the other two points were a primary tumor lesion in the prostate and a section of urine catheter *in vitro* according to their position. Therefore, except for the tracing of bone metastatic lesion, the utilization of detecting lymph node of metastasis by the probe of ^18^F-Alfatide II should also be available. **(C)** The direction cross focused on the metastatic lesion in the lumbar 1 vertebra. The first, second, and third rows corresponded to the view of regular CT, PET-CT (^18^F-Alfatide II), and mergence in three directions in the lesion of the lumbar 1 vertebra. The first row presented the high density of bone cortex in the lumbar 1 vertebra, which also tallied with the feature of bone metastasis in PCa. The second row showed the abnormal high intake lesion of ^18^F-Alfatide II in the lumbar 1 vertebra, hinting the malignant tendency of this lesion. As the mergence of the first and second rows, the third row displayed the abnormal lesion in the lumbar 1 vertebra through high brightness signal. **(D)** The direction cross focused on the metastatic lesion behind the left ilium. The first, second, and third rows separately revealed the observed lesion behind the left ilium in the view of regular CT, PET-CT (^18^F-Alfatide II), and combination of regular CT and PET-CT in three directions. In the first row, we could find the unusual bone hyperplasia behind the left ilium. Then, the high intake of tracer in the views of PET-CT (^18^F-Alfatide II) revealed malignant metastatic lesion. The third row provided the combination of the first and second rows that we could discover corresponding region of another bone metastatic lesion.

There was not any report of any side effects following the injected dose of ^18^F-Alfatide II in the participates. No adverse events were noted during the imaging. We displayed the representative cases that could demonstrate bone metastases simply and intuitively. For the 82-year-old man, the ^18^F-Alfatide II was concentrated in local lesion while the abnormities in the left side of the middle part of the pubis and the right side of the lumbar 1 vertebra were also observed (**Figure 8**). For another 76-year-old man, the ^18^F-Alfatide II was high intake in right side of the external iliac vessel, the lumbar 1 vertebra and behind the left ilium, together with the prostate (**Figure 9**). These outcomes hinted the research of integrin αvβ_3_, combined with other techniques, had widely applied prospect in clinical practice.

## Discussion

PCa cells possess a propensity to metastasize to bone (1). The bone metastasis microenvironment is a requirement for the interactions between PCa cells and bone cells (43). Therefore, it is crucial to identify novel metastasis-related matricellular proteins. Our findings highlighted pro-metastasis role of POSTN in PCa bone metastasis. It was observed that the expression of POSTN was high in PCa, especially highest in PCa metastasized to bone. Importantly, inhibiting POSTN in PCa cells resulted in a significant reduction of metastatic PCa cells in bone. Taken together, targeting POSTN in PCa cells as well as in the osteoblastic may be an effective treatment for PCa bone metastasis.

In this study, we summarized previous studies on POSTN, and postulated that POSTN was a mediator in forming the positive feedback loop between PCa and osteoblasts. When the dissociative PCa cells transfer to bone with the blood flow, they secrete POSTN to stimulate the proliferation of osteoblasts. The proliferative osteoblasts also secrete more POSTN to promote the proliferation and invasion of PCa cells. This positive feedback loop between PCa and osteoblasts results in local enrichment of POSTN and a favorable “soil” for the settlement and growth of the “seeds.” The data validated this hypothesis. We found PCa cell-derived POSTN promotes proliferation of osteoblasts and osteoblast-derived POSTN promotes proliferation and invasion of PCa cells. Further observation showed that POSTN knockdown could suppress the capability of bone metastasis in PCa cells *in vivo*.

One of the highlights of this study is the usage of multiomics. We built a full network in both the upstream and downstream of POSTN by Metacore™ and IPA while we confirmed the connection between POSTN and bone metastasis in PCa. Besides the finding of TGF-β1/POSTN/integrin αvβ3 axis in PCa bone metastases, we also found that, in the downstream of integrin αvβ_3_, there were two key proteins including c-Src (44) and MMP-2 (45), and meanwhile, the function of integrin αvβ_3_ was regulated by Tenascin-C (46), which are extremely involved in metastasis. This network predicted a potential domain for further investigation. Through the network of IPA, we explored a series of upstream transcript factors. The family numbers of TEAD are, for example, the transcription factors that function to modulate gene expression in regulation of cell growth, cell proliferation, and organ development by the Hippo signaling pathway (47). The interactive relationship between the Hippo pathway and the tumor microenvironment could drive tumor progression and metastasis in PCa (48). These findings provide new ideas to explore the regulation of POSTN.

Another highlight of this study is the involvement of ^18^F-Alfatide II as the molecule probe of integrin αvβ3 in PET-CT detection. Although bone scan with ^99^mTc-phosphonates is widely used for the evaluation of bone metabolism in patients with PCa, because of false positive findings in benign conditions like inflammation and arthritis, the specificity of ^99^mTc-phosphonates only ranges from 60-75% (49). Therefore, newer tracers are needed to develop for detecting accuracies for small, incipient metastatic foci in PCa (50). To discovery the clinical application, ^18^F-Alfatide II which is a high-efficient tracer was applied to detect the suspicious lesions in patients of PCa. Since the high tumor accumulation is the most important property of the optical imaging tracer, the usage of ^18^F-Alfatide II revealed a high sensitivity and specificity to identify the bone metastases. Nevertheless, the more clinical implications of this tracer for detecting disease foci still demands systematized evaluation.

In conclusion, our findings suggested that POSTN provided a favorable microenvironment for the proliferation and invasion of PCa cells and the proliferation of osteoblasts, and thus may play an important role in osteoblastic metastasis of PCa (51). Furthermore, the principal role of POSTN in establishing and remodeling of the metastatic niche for PCa provided a promising clinical application for early diagnosis, effective treatment, and even prevention.

## Data Availability Statement

The original contributions presented in the study are included in the article/**Supplementary Material**. Further inquiries can be directed to the corresponding authors.

## Ethics Statement

The studies involving human participants were reviewed and approved by the Huashan Institutional Review Board. The patients/participants provided their written informed consent to participate in this study. The animal study was reviewed and approved by the Huashan Institutional Review Board. Written informed consent was obtained from the owners for the participation of their animals in this study.

## Author Contributions

YZ analysis all data in the paper. G-WX, C-YS and Y-YM designed the program. Q-FH, Y-FT, KX and Y-JG contributed data acquisition and analysis tools. S-YG wrote the draft paper. FZ revised the manuscript and secured the funding. All authors read and approved the final manuscript.

## Funding

This work was supported by the National Key R&D Program of China (2021YFF0703702), National Natural Science Foundation (81802576), Wuxi Commission of Health and Family Planning (ZM001), National Natural Science Foundation of China (No. 81201620 and 81372316) and Chen Guang Program of Shanghai Municipal Education Commission (N158554).

## Conflict of Interest

The authors declare that the research was conducted in the absence of any commercial or financial relationships that could be construed as a potential conflict of interest.

## Publisher’s Note

All claims expressed in this article are solely those of the authors and do not necessarily represent those of their affiliated organizations, or those of the publisher, the editors and the reviewers. Any product that may be evaluated in this article, or claim that may be made by its manufacturer, is not guaranteed or endorsed by the publisher.

## Supplementary Material

Supplementary Figure 1 Osteoblasts induce POSTN expression in PC cells via TGF-β. **(A)** MDA PCa 2b cells were incubated with 30% OBCM and the expression of POSTN was detected by real-time PCR. **(B)** MDA PCa 2b cells were incubated with 30% OBCM and the expression of POSTN was detected by western blotting. **(C)** MDA PCa 2b cells were incubated with 30% OBCM and an inhibitor of TGF-β signaling (LY364947). The expression of POSTN was detected by real-time PCR. **(D)** MDA PCa 2b cells were incubated with 30% OBCM and an inhibitor of TGF-β signaling (LY364947). The expression of POSTN was detected by western blotting. *p < 0.05 compared with control; ^#^p < 0.05 compared with the OBCM or PCCM-treated group.
