# Supplementary figures and images for: Tumor- and Osteoblast-Derived Periostin in Prostate Cancer bone Metastases
Source: Front Oncol. 2022 Jan 11;11:795712. doi: 10.3389/fonc.2021.795712 (PMC8787093; doi:10.3389/fonc.2021.795712)

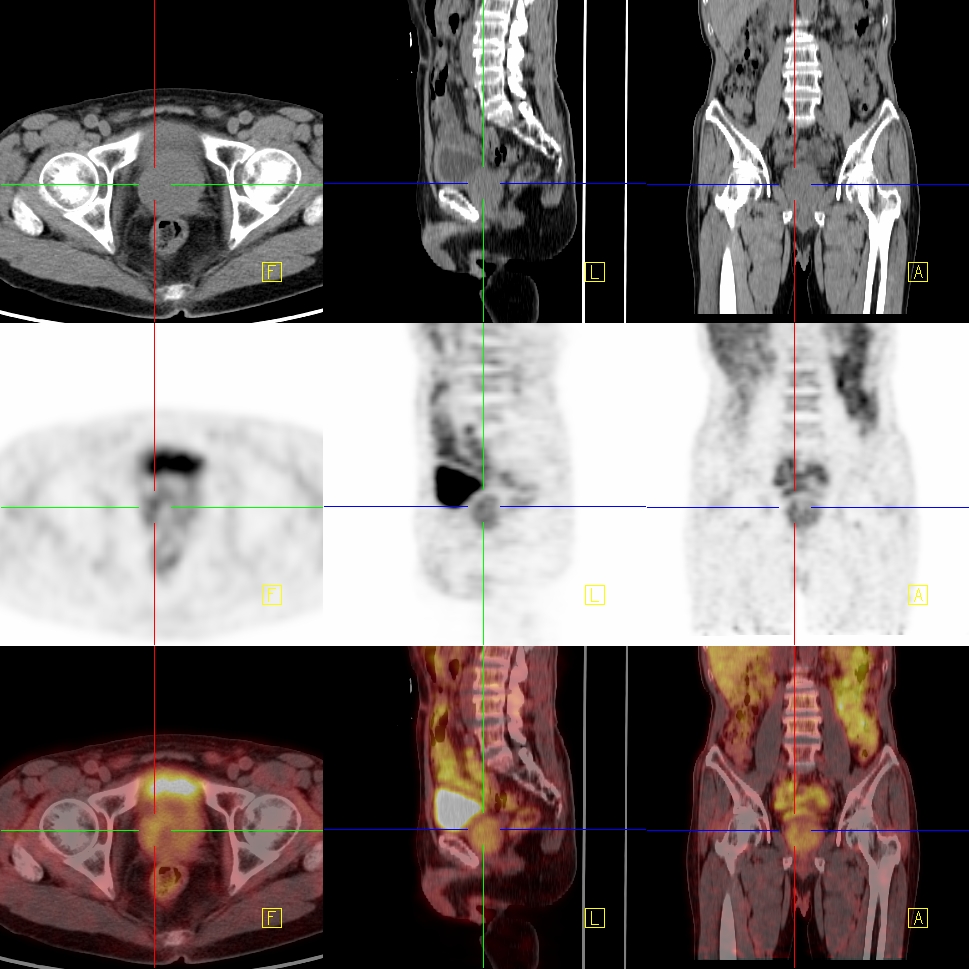

Supplement: Supplementary Figure 1 — Osteoblasts induce POSTN expression in PC cells via TGF-β. (A) MDA PCa 2b cells were incubated with 30% OBCM and the expression of POSTN was detected by real-time PCR. (B) MDA PCa 2b cells were incubated with 30% OBCM and the expression of POSTN was detected by western blotting. (C) MDA PCa 2b cells were incubated with 30% OBCM and an inhibitor of TGF-β signaling (LY364947). The expression of POSTN was detected by real-time PCR. (D) MDA PCa 2b cells were incubated with 30% OBCM and an inhibitor of TGF-β signaling (LY364947). The expression of POSTN was detected by western blotting. *p < 0.05 compared with control; #p < 0.05 compared with the OBCM or PCCM-treated group. [file Image_1.jpg]

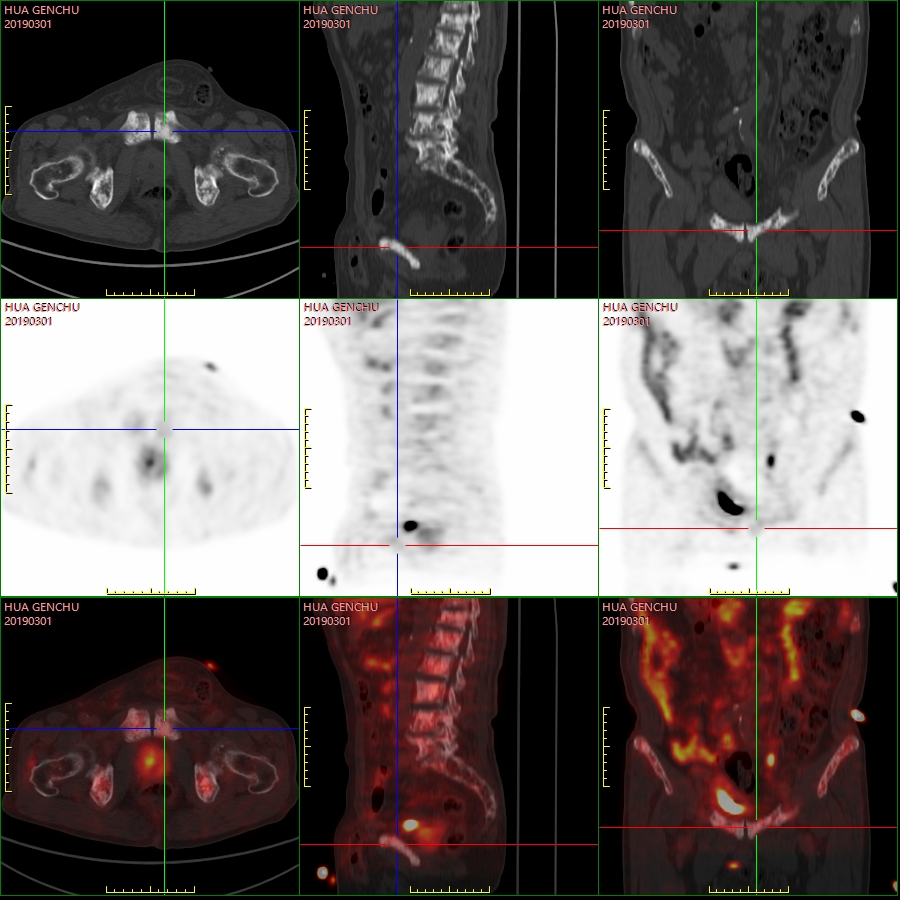

Supplement: Supplementary file 2 [file Image_2.jpg]

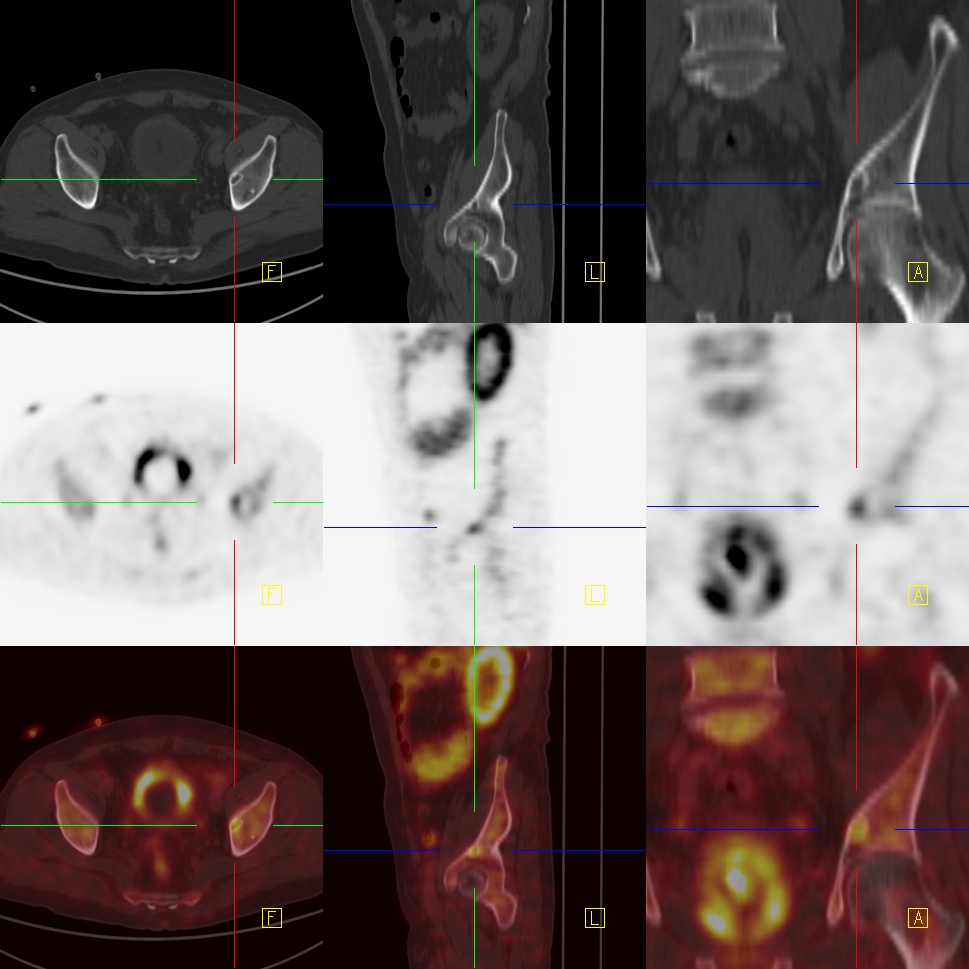

Supplement: Supplementary file 3 [file Image_3.jpg]

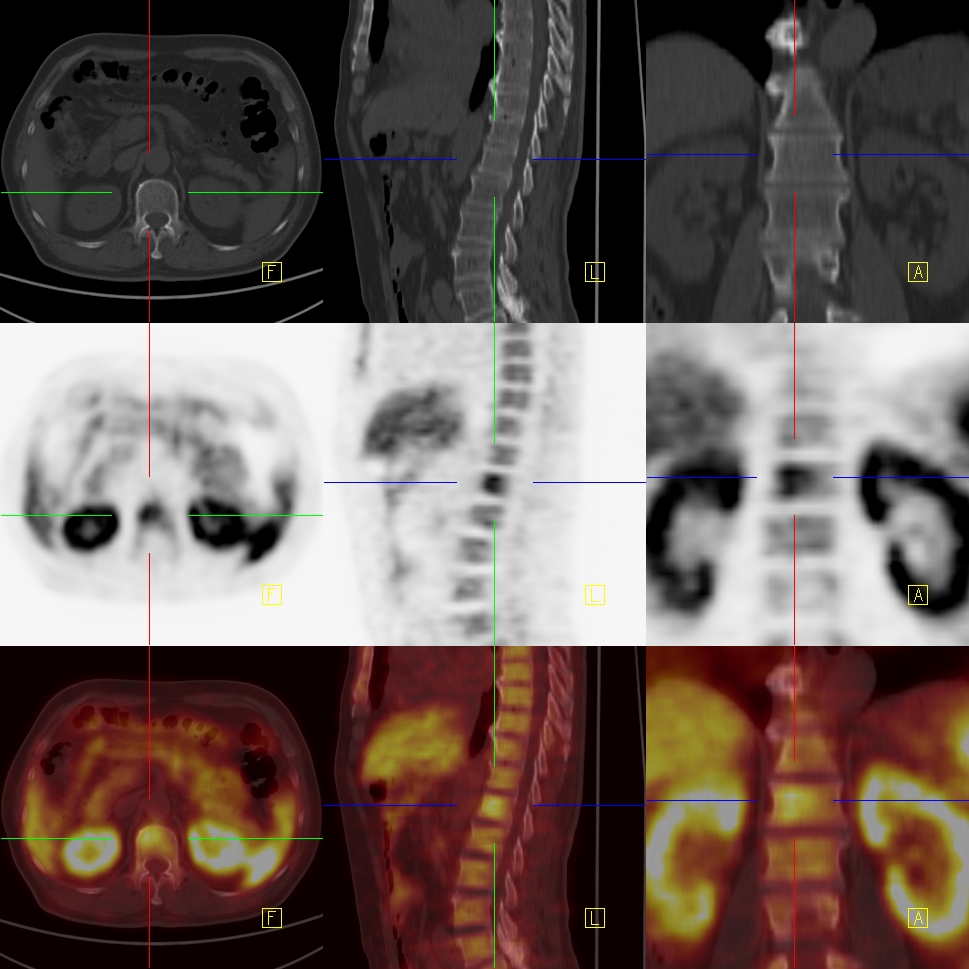

Supplement: Supplementary file 4 [file Image_4.jpg]

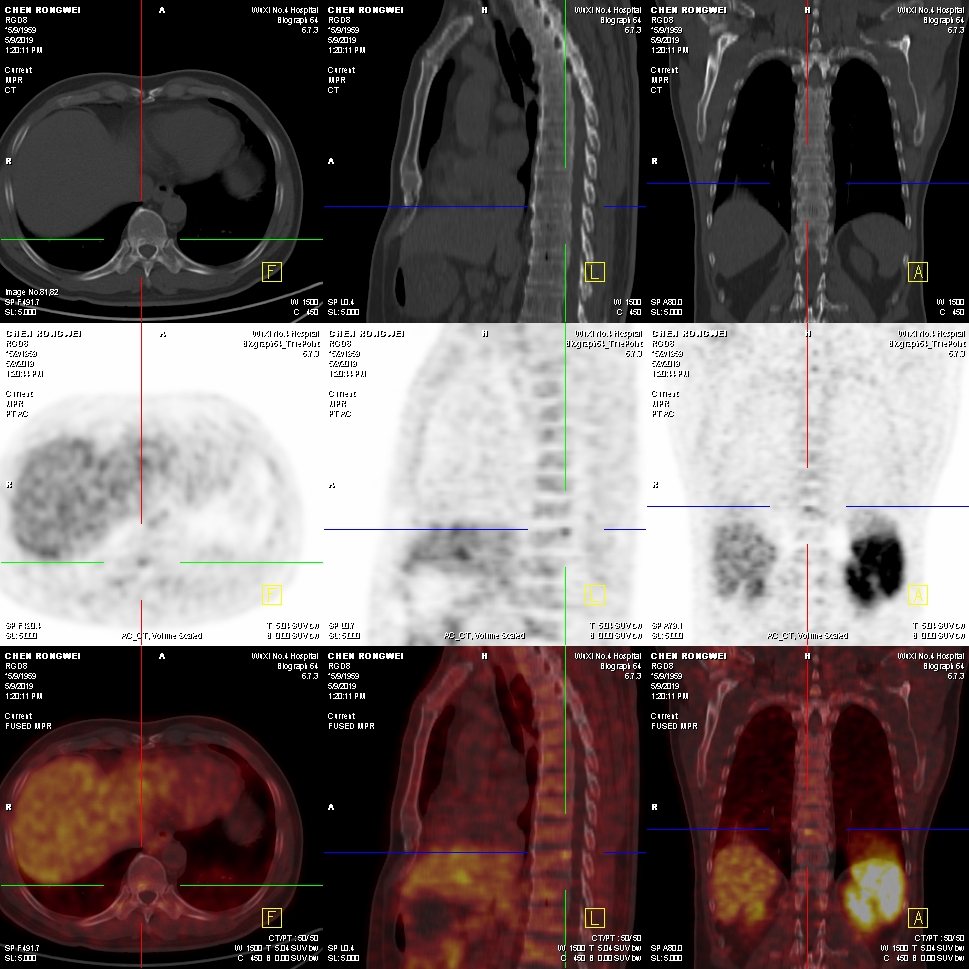

Supplement: Supplementary file 5 [file Image_5.jpg]

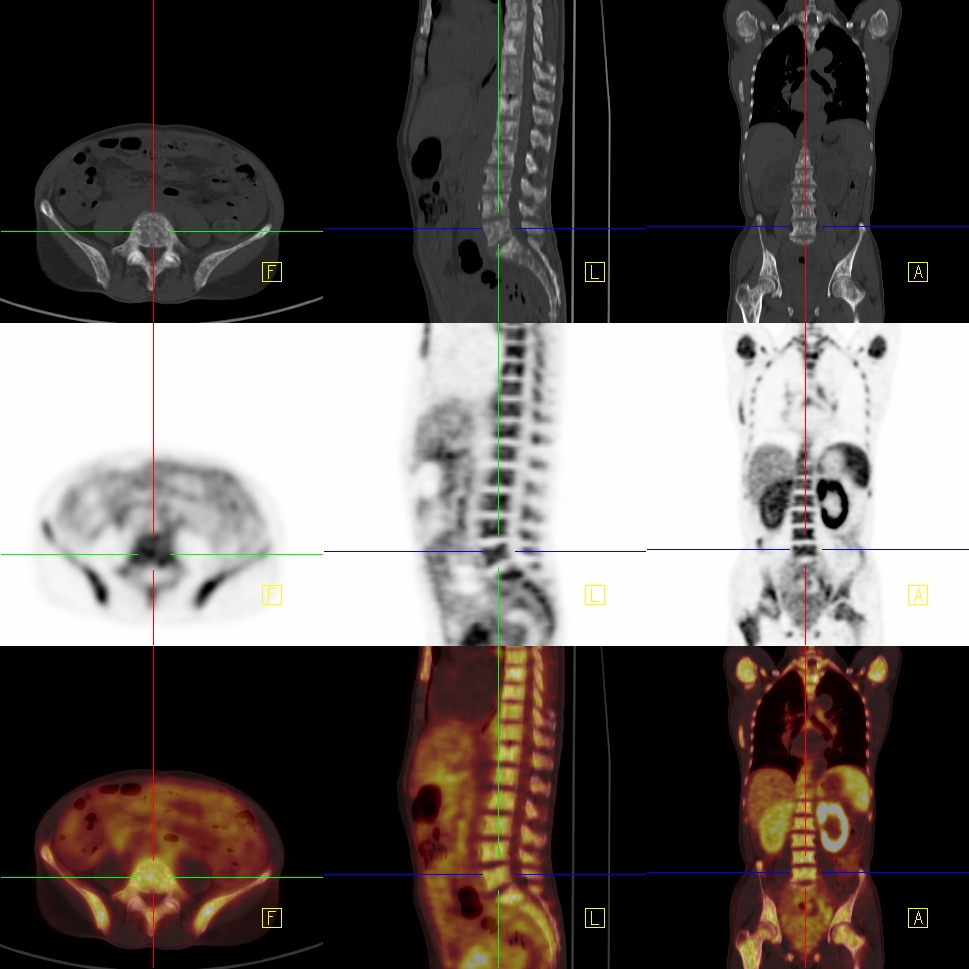

Supplement: Supplementary file 6 [file Image_6.jpg]

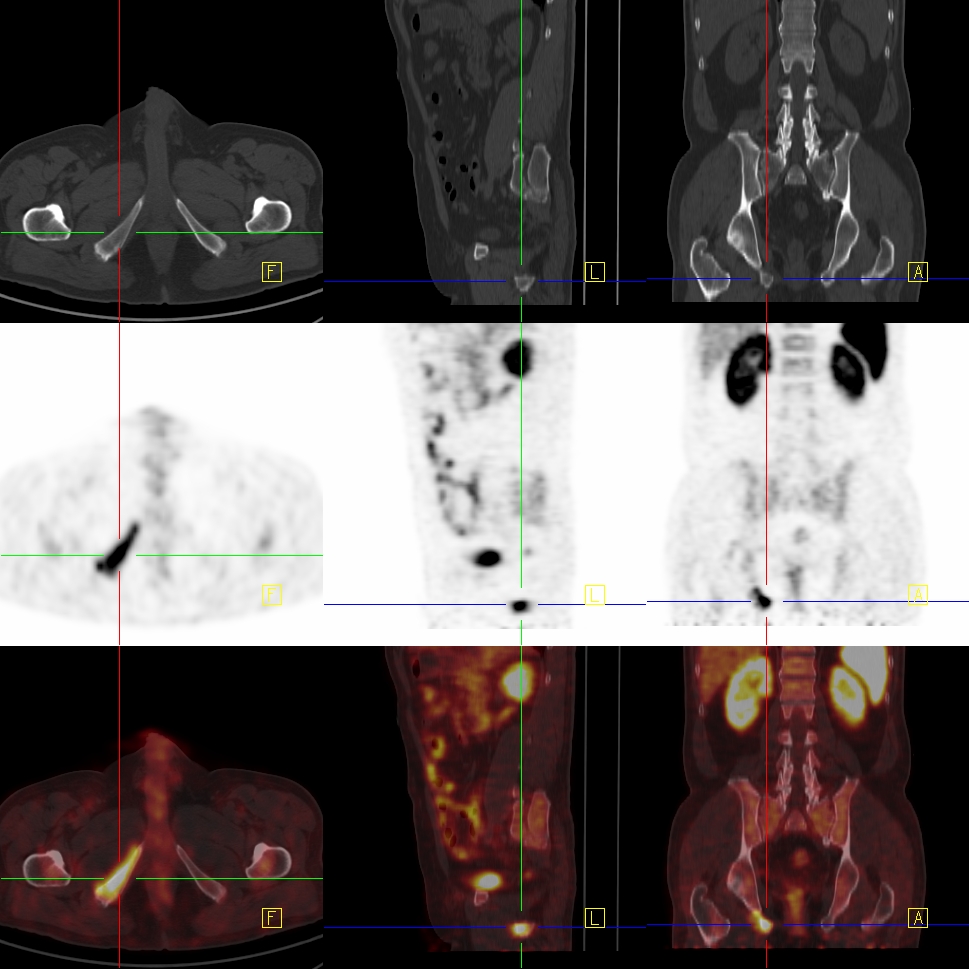

Supplement: Supplementary file 7 [file Image_7.jpg]

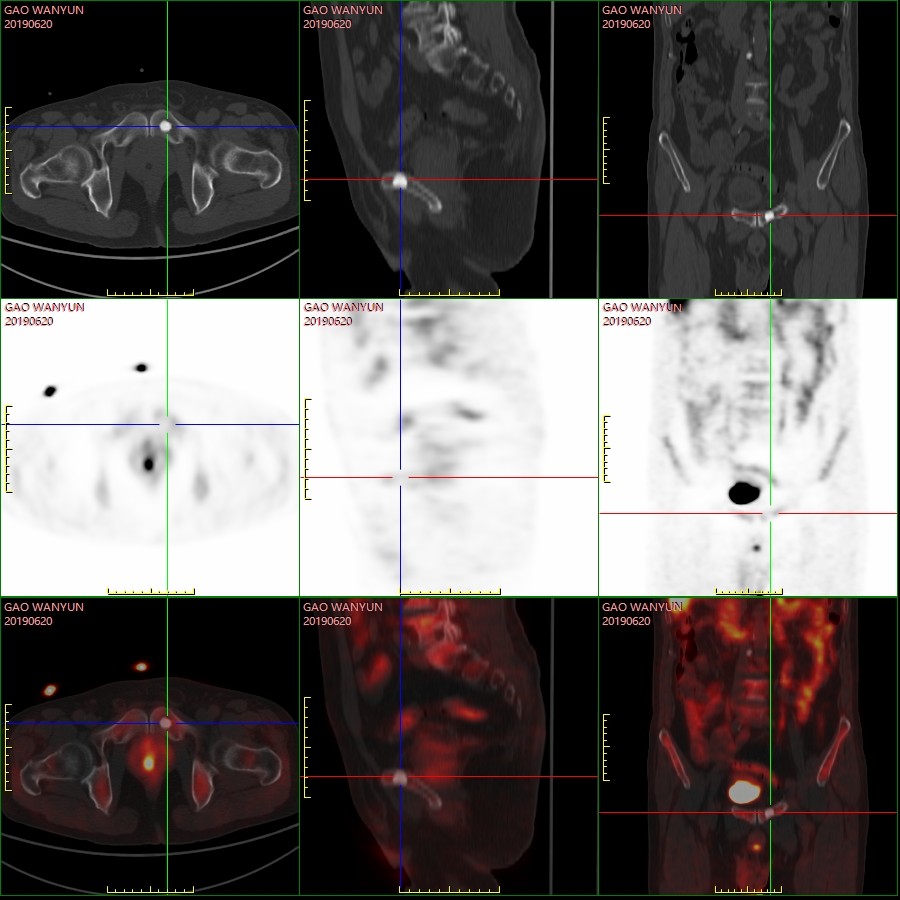

Supplement: Supplementary file 8 [file Image_8.jpg]

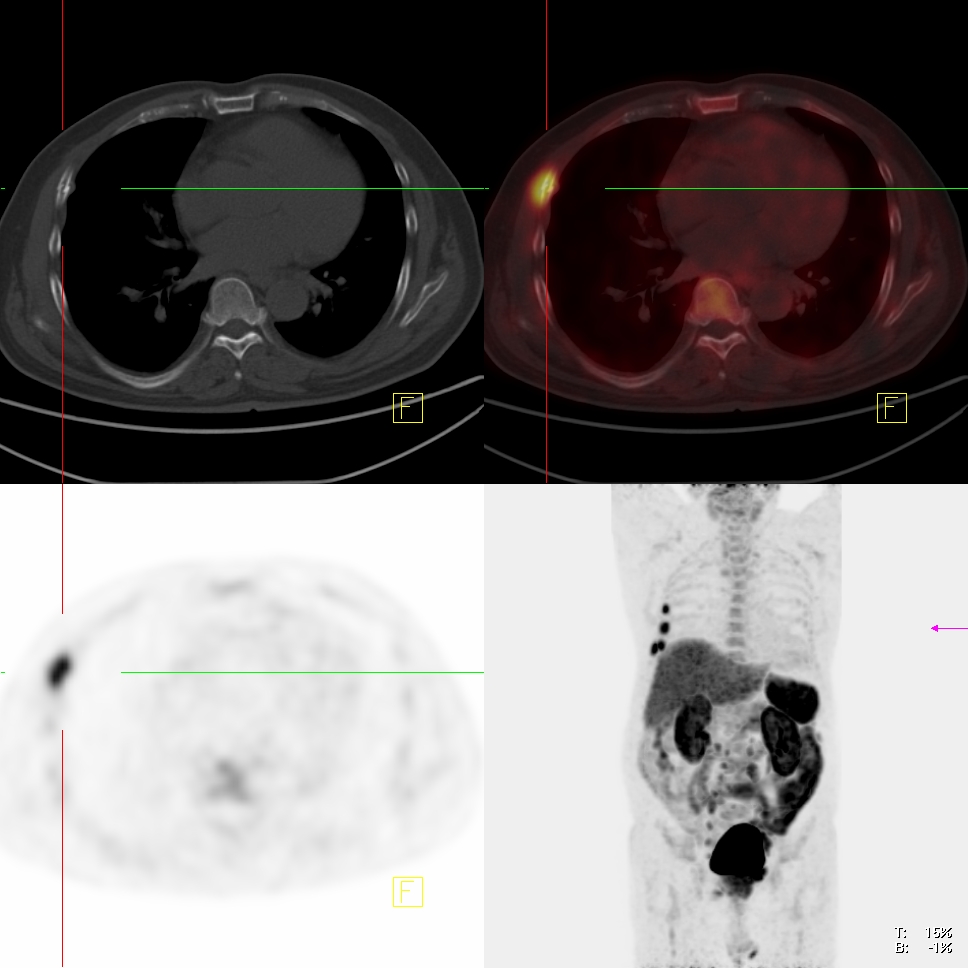

Supplement: Supplementary file 9 [file Image_9.jpg]
